# Understanding Social Anxiety Disorder in Adolescents and Improving Treatment Outcomes: Applying the Cognitive Model of Clark and Wells (1995)

**DOI:** 10.1007/s10567-018-0258-5

**Published:** 2018-04-13

**Authors:** Eleanor Leigh, David M. Clark

**Affiliations:** 1 Department of Experimental Psychology, University of Oxford, Oxford, UK; 2 Oxford Centre for Anxiety Disorders and Trauma, The Old Rectory, Paradise Square, Oxford, OX1 1TW UK

**Keywords:** Social anxiety disorder, Adolescents, Young people, Cognitive model, Cognitive therapy, Psychological therapy

## Abstract

Social anxiety disorder is a condition characterised by a marked and persistent fear of being humiliated or scrutinised by others. Age-of-onset data point to adolescence as a developmentally sensitive period for the emergence of the condition, at a time when the peer group becomes increasingly important. Social anxiety in adolescence is associated with considerable impairment that persists through to adulthood. There are clear potential benefits to delivering effective interventions during adolescence. However, there is limited evidence on the specific efficacy of available therapies. This is in contrast to adults, for whom we have interventions with very specific treatment effects. One such treatment is individual cognitive therapy. Cognitive therapy is based on the cognitive model of social anxiety proposed by Clark and Wells (in: Heimberg, Leibowitz, Hope, Scheiber (eds) Social phobia: diagnosis, assessment and treatment, The Guilford Press, New York, 1995). The present review examines the potential application of this adult cognitive model to the understanding of adolescent social anxiety and considers additional adolescent-specific factors that need to be accommodated. It is suggested that a developmentally sensitive adoption of the cognitive model of social anxiety disorder (Clark and Wells 1995) for adolescents may lead to better treatment outcomes.

## Introduction

Social anxiety disorder (SAD) is a debilitating condition characterised by a marked and persistent fear of being humiliated or scrutinised by others (World Health Organization 1992; American Psychiatric Association 2013). Individuals fear a range of social interactions, such as conversations with strangers, joining in groups or speaking on the telephone. Most things that involve being observed by others are difficult. These include walking into a room when other people are already seated, eating or drinking in public, and performing in front of an audience. Sufferers fear that they will say or do something that they believe will be humiliating or embarrassing. Common concerns include the fear of sweating, shaking, blushing, stumbling over words, looking anxious, or appearing boring, stupid, or incompetent (Stein and Stein 2008).

Social anxiety disorder is the third most common mental health disorder after depression and substance abuse, with lifetime prevalence rates of around 12% (Kessler et al. 2005). It is common in young people. Prevalence rates of around 10% have been reported by the end of adolescence in US and New Zealand samples (Burstein et al. 2011; Merikangas et al. 2010; Feehan et al. 1994). Social anxiety disorder persists in the absence of treatment (Bruce et al. 2005; Reich et al. 1994a, b). For example, Bruce et al. (2005) reported findings of a US-based community study in which adults with various anxiety disorders were followed up for 12 years. At the start of the study, individuals had suffered with social anxiety disorder for 19 years on average, and over the next 12 years only 37% recovered. This is compared with recovery rates of 58% for generalised anxiety disorder and 82% for panic disorder without agoraphobia. Remarkably similar findings have been reported in adolescent samples. A 10-year prospective community study with over 3000 German adolescents and young adults (aged 14–24 years) (Beesdo-Baum et al. 2012) found that 57% of those with social anxiety disorder at the start of the study were still reporting at least symptomatic social anxiety at follow-up and higher persistence of social anxiety was significantly predicted by an earlier age of onset of the disorder. Only 15% were completely remitted.

Social anxiety disorder is associated with profound negative consequences and high levels of impairment even when compared to other psychiatric disorders (Alonso et al. 2004). Social anxiety disorder affects all areas of life. For adolescents, academic attainment is curtailed, with individuals at risk of leaving school early and obtaining poorer qualifications (Van Ameringen et al. 2003). Amongst a sample of 784 Finnish 13–17-year olds, those with clinical or subclinical social anxiety disorder had a lower grade point average compared to those with no diagnosis (Ranta et al. 2009). Social relationships are inevitably particularly challenging for socially anxious adolescents. They report having fewer friends, and the peer and romantic relationships they do have are of poorer quality (La Greca and Harrison 2005; Hebert et al. 2013). They are more likely to be victims of bullying (Acquah et al. 2016; Ranta et al. 2009). Social anxiety makes day-to-day life difficult, for example, shopping and using the telephone can be a challenge. Research with adults demonstrates that the impairments associated with social anxiety disorder persist into adulthood. Employment is affected: although the majority of adults with social anxiety disorder are employed, they take more days off and report lower productivity due to their symptoms (Stein and Kean 2000). In terms of close relationships, adults with social anxiety disorder are less likely to marry, more likely to divorce and less likely to have children (Wittchen et al. 1999a). A study amongst adults seeking treatment for social anxiety disorder found that the functional impairment was not due to the presence of comorbid mood or anxiety disorders (Aderka et al. 2012).

## Adolescence and the Development of Social Anxiety Disorder

Social anxiety is very much a disorder with its origins in adolescence, with the majority of cases occurring during this period (90% occur by the age of 23 years; Kessler et al. (2005)). Prospective, longitudinal studies suggest that it is relatively unusual in early childhood (Wittchen et al. 1999b), with incidence increasing through the adolescent years and a median age of onset of 13 years (Kessler et al. 2005). After this peak period of onset, new cases are fairly rare after about the age of 25 years (Heimberg et al. 2000). The increased incidence of social anxiety disorder during adolescence is perhaps unsurprising. Adolescence is a time when people are moving from a unique reliance on their family unit and are learning how to interact with peers in a way that will set them up for the rest of their life. They become increasingly autonomous from their parents and reliant instead upon their peer group (Larson and Richards 1991).

Underpinning this social reorientation is the development of particular neurocognitive abilities (Kilford et al. 2016). One of these is self-consciousness. Self-consciousness is the directing of attention inwards, with both a private and public dimension (Davis and Franzoi 1999). Private self-consciousness refers to an awareness of one’s inner thoughts and feelings, whilst public self-consciousness is an awareness of the self as a social object. Self-consciousness, and particularly the public aspect of it, is thought to peak in early adolescence (Cicchetti and Cohen 2006; Rankin et al. 2004; Vartanian 2000). The development of public self-consciousness will enhance adolescents’ sensitivity to how they are being perceived by others. This awareness will inform how adolescents behave towards other people and will help them to establish more mature and enduring relationships with their peers. However, it seems very plausible that an acute awareness of the self as a social object may also confer vulnerability for increased social anxiety and in line with this suggestion, public self-consciousness has been found to be related to social anxiety in adolescents (Mallet and Rodriguez-Tomé 1999) and in adults (Mor and Winquist 2002). Although all young people seem to experience a normative increase in public self-consciousness in early adolescence, only a small proportion will develop persistent social anxiety, and so it is not in itself enough to explain the increased incidence. Rather, self-consciousness may be a necessary precursor implicated in the development and maintenance of social anxiety (Tillfors and Van Zalk 2015). It seems plausible that the acute self-consciousness experienced during early adolescence renders this a developmentally sensitive period for the emergence of social anxiety (Haller et al. 2015).

As well as heightened self-consciousness, adolescence is also normally a period of strong sensitivity to peer influence and it is a crucial phase of social learning. Social relationships during adolescence are especially rewarding during this time, and it has been suggested that this increases the impact of both positive and negative aspects of social interactions (Kilford et al. 2016). In line with this, studies consistently demonstrate that peer rejection leads to increased distress, anxiety and lower mood in adolescents compared to children and adults (Platt et al. 2013). The heightened emotional salience of peer interactions means adolescents are primed to prioritise the development of social networks, but for some it will also bring about an increased vulnerability for the emergence and maintenance of social fears (Eldreth et al. 2013).

In adulthood we pay attention to what others think, but there is a certain resistance to peer influence. In contrast during adolescence, this resistance is far weaker, with young people showing a strong susceptibility to peer influence. This can present as conformism to trends. A study examining the effects of peer influence on risk taking in adolescents and peers bears this suggestion out (Gardner and Steinberg 2005). When playing a driving-based video game, youths took significantly more risks when with peers than when alone, whereas adult risk-taking behaviour was not affected by the presence of peers. Studies looking at resistance to peer influence are consistent with this finding. At the beginning of adolescence, this resistance is low and only gradually increases to adult levels (Steinberg and Monahan 2007). Susceptibility to peer influence in adolescence is likely to be adaptive, as it will provide the opportunity to form strong social bonds and learn vital lessons about relationships. However, it is also like to represent a developmental sensitivity for the emergence of social fears.

As this brief discussion highlights, adolescence ushers in a host of changes at various levels including in neural circuitry, information processing and the social environment (Blakemore 2008). For the majority of adolescents, one of the consequences of these changes is a short-lived increase in social fears (Weems and Costa 2005). But for a subset, perhaps those who are more behaviourally inhibited by temperament, it has been proposed that these cognitive, brain maturational and social changes confer vulnerability for the development and also the maintenance of social anxiety disorder (Caouette and Guyer 2014). With this in mind, any theoretical accounts that aim to explain the persistence of social anxiety in adolescence need to be positioned within a developmental context. In other words, it is necessary to take into account developmental influences on maintenance processes that are relevant across the lifespan, as well as considering processes that may be unique to the adolescent period.

When considering treatment, it is clear that understanding and intervening in social anxiety disorder in adolescence is vital in order to avert long-term consequences. But in addition to this, the plasticity associated with adolescence may also offer a ‘window of opportunity’ in which to provide particularly potent interventions (Haller et al. 2015).

## Current Treatments for Social Anxiety Disorder in Adolescents

Traditionally, cognitive behavioural therapies (CBT) did not target specific anxiety disorders in young people. Instead, social, separation and generalised (or overanxious) anxiety disorder were all treated with the same set of techniques. Creswell et al. (2014) suggest this approach was motivated by two principal factors. Firstly, the high comorbidity amongst the anxiety disorders in children and young people and secondly, the lack of well-validated disorder-specific maintenance models. The most well-known examples of the ‘generic’ CBT approach are ‘Coping Cat’ for children (Kendall and Hedtke 2006) and the ‘CAT Project’ for adolescents (Kendall et al. 2002). The treatments usually comprise 16 sessions and involve a combination of psycho-education, anxiety management strategies and graded exposure. There have been many large randomized controlled trials undertaken examining the effectiveness of Coping Cat and its various relations in treating separation, social and generalised anxiety disorder (e.g. Ginsburg et al. (2011), Walkup et al. (2008)). Meta-analyses have shown that these treatments are associated with substantial effect sizes (Bennett et al. 2013). However, a number of studies have shown that outcomes from generic CBT are less good for young people with SAD compared to those with other anxiety disorders. Young people (mixed child and adolescent samples) with SAD are significantly less likely to lose their diagnosis of SAD after treatment compared with young people with other anxiety diagnoses (Crawley et al. 2008; Ginsburg et al. 2011; Hudson et al. 2015a, b; Lundkvist-Houndoumadi and Thastum 2017; Kodal et al. 2018).

As well as traditional generic CBT, psychological therapies designed specifically for social anxiety disorder have been developed. Cognitive behavioural group therapy (CBGT; Albano et al. (1995)) was one of the first to be tested. This treatment, based on the Heimberg model (Rapee and Heimberg 1997), involves psycho-education and skills training (social skills and anxiety management strategies) followed by exposure tasks (Albano and DiBartolo 2007). In an early randomized controlled trial (RCT), CBGT was compared to no treatment amongst female adolescents (Hayward et al. 2000). The authors note that whilst those in the CBGT group showed significantly greater reductions in social anxiety symptoms compared to the no treatment group post-treatment, they continued to report considerable residual symptoms and at one-year follow-up the control group had also improved and there was no longer a significant group difference. Herbert et al. (2009) did not find evidence for specific treatment effects of CBGT in an RCT comparing the treatment to an educational supportive therapy amongst adolescents, with both treatments associated with an improvement in social anxiety symptoms, functioning and social skills.

Social Effectiveness Therapy (SET; Turner et al. (1994)) and its parallel version for 8–12-year olds Social Effectiveness Therapy for Children (SET-C; Beidel et al. (2000)) is another SAD specific treatment. It is a behavioural group treatment comprising psycho-education, social skills training and exposure. Baer and Garland (2005) adapted the treatment for adolescents and compared it to waitlist in a pilot RCT with 12 adolescents. SET-C outperformed waitlist based on clinician- and self-report of symptoms. Olivares et al. (2002) compared SET-C and CBGT to a waitlist control using a quasi-experimental design (participants were allocated to group according to their school timetables; random allocation was not used). Both active treatments yielded significantly better self- and clinician-rated improvements compared to waitlist with no differences between the two, and gains were maintained at 5-year follow-up (Garcia-Lopez et al. 2006).

Masia-Warner and colleagues (2001) adapted SET-C for an adolescent schools-based population, and they have tested the treatment, Skills for Academic Success (SASS) in two RCTs. Compared to waitlist control, SASS led to significantly greater improvements in social anxiety, functioning and social skills based on self-report, parent-report and clinician-report (Masia-Warner et al. 2005). Examining specific treatment effects, the authors compared SASS with an attention control which involved psycho-education and supportive therapy (Masia-Warner et al. 2007). Findings showed significantly greater improvement in clinician-report and self-report but not parent-report of social anxiety after SASS compared to the comparison, suggesting preliminary evidence for treatment specificity. Whilst results from these two studies are very encouraging, because SASS was designed to be a low-intensity school-based intervention, the findings may not be directly relevant to the treatment of clinically referred adolescents.

Overall, the literature on specific CBT-based interventions for social anxiety disorder in *adolescents* show that a number of interventions are effective compared to no treatment; however, only one study has provided evidence for treatment specificity, in the sense of being superior to other credible interventions.

## Improving Treatment Outcomes

In contrast to the limited evidence for treatment specificity in adolescents, substantial evidence for specific treatment effects has been observed in adults (Mayo-Wilson et al. 2014), including CBT treatment based on the Heimberg model (Rapee and Heimberg 1997). The version of CBT for adults that has the most evidence for treatment specificity is a specialised form of individual cognitive therapy, developed to target the cognitive abnormalities and maintenance processes specified in the Clark and Wells (1995) model of social anxiety disorder. Whilst there is overlap between this model and other cognitive behavioural models of social anxiety (Wong and Rapee 2016) and the treatment has similarities to some other CBT approaches, many of the techniques are distinct. This can be seen in Table 1 which summarises what does and does not happen in cognitive therapy for social anxiety disorder.

**Table 1:** Summary of what does and does not happen in cognitive therapy for social anxiety disorder

What happens in cognitive therapy
Focuses on targets specified in Clark and Wells (1995) model
Personal version of model
Experiential exercise to demonstrate adverse effects of self-focused attention and safety behaviours
Video (and still) feedback to correct negative self-images
Attention training to promote external focus
Behavioural experiments to test patients’ fearful predictions in social situations whilst dropping safety behaviours and/or enacting feared outcomes
Surveys to discover other people’s view of feared outcomes
Memory work (discrimination training and memory rescripting) to reduce impact of early social trauma

What does not happen in cognitive therapy
Repeated exposure to promote habituation
Exposure hierarchies
Rating anxiety in feared situations (SUDS)
Thought records
Rehearsal of rationale responses in social situations (self-instruction)
Social skills training

Cognitive therapy based on the Clark and Wells (1995) has been compared to a number of other active treatments, and in the six randomized controlled trials that have been undertaken it has been shown to be superior to: Group CBT (Stangier et al. 2003; Mörtberg et al. 2007), exposure therapy (Clark et al. 2006), interpersonal psychotherapy (Stangier et al. 2011), psychodynamic psychotherapy (Leichsenring et al. 2013), fluoxetine (Clark et al. 2003), medication-based treatment as usual (Mörtberg et al. 2007) and pill placebo (Clark et al. 2003). Therapy based on the Clark and Wells (1995) model is one of the two first-line treatments recommended by the National Institute for Health and Care Excellence (NICE 2013), an independent body that synthesises available research evidence to develop treatment guidelines [the other recommended treatment being individual CBT based on the Heimberg model (1997)].

Such consistent and broad evidence for treatment specificity is unusual and suggests that it might be profitable to investigate whether the Clark and Wells’ (1995) model applies to adolescent social anxiety disorder as well as the adult condition. If the model does apply to adolescents, then a treatment that rather single-mindedly focuses on the maintenance factors specified in the model may be helpful for this population. Certainly, this type of approach, in which interventions are very tightly tied to known maintenance factors, has proved successful as a strategy for developing effective forms of CBT for a range of anxiety-related conditions in adults (Clark 2004).

## Clark and Wells’ Cognitive Model of Social Anxiety in Adults

Socially anxious individuals will face many social situations every day, and the vast majority of these are benign, so why does social anxiety persist? A number of cognitive accounts have been put forward to try to explain this (Clark and Wells 1995; Heimberg et al. 2010; Hofmann 2007; Rapee and Heimberg 1997). There is considerable overlap amongst these models, for example they all highlight the importance of fear of negative evaluation and of self-focused attention in maintaining social anxiety. A useful review of the prominent cognitive behavioural models including a description of their commonalities and differences is provided by Wong and Rapee (2016).

**Fig. 1:**
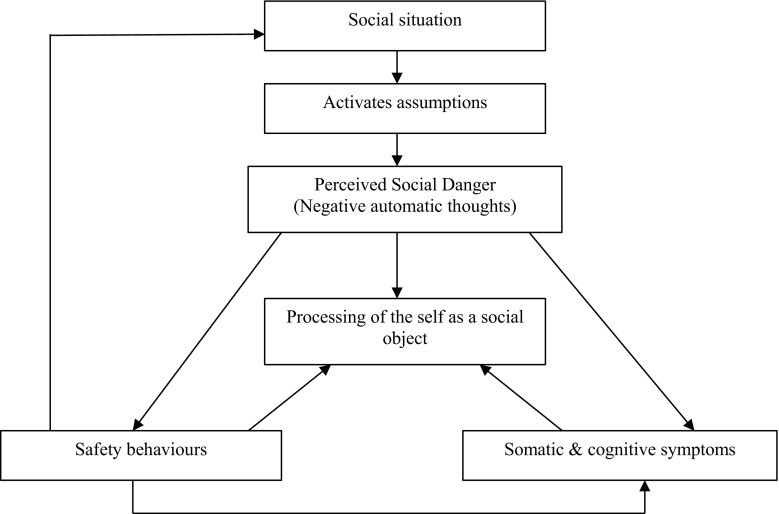
Cognitive model of social anxiety disorder (Clark and Wells 1995)

According to the cognitive model developed by Clark and Wells (1995), people with social anxiety hold firm beliefs about the importance of making a good impression to others, but they also believe they come across badly (Leary 2001). Broad unconditional beliefs such as ‘I am weird’ lead them to make assumptions about themselves and their social environment. These usually involve high self-expectations (“I must always look cool and calm”) and conditional beliefs about their social behaviour (“If I look at all anxious people will think I am a gibbering wreck”) (Wong and Moulds 2011). These negative beliefs are activated in social settings and understandably trigger alarm (Hofmann 2007). The sense of threat then motivates a chain of cognitive, affective and behavioural responses. This chain of responses is self-perpetuating and closed off to new information. Several inter-linked processes are emphasised in the model: a shift to an internal focus of attention and the use of internal information to infer how one appears to others (collectively described as ‘processing of the self as a social object’); safety behaviours; and worry and rumination that occur before and after the social event. These are described in more detail below, and the model is displayed in Fig. 1.

First, the model suggests that when individuals enter a social situation their attention will shift to a predominantly internal focus, in order to closely monitor how they are coming across. One of the reasons that this self-focused attention is problematic is because it reduces the opportunity for the individual to process the social situation and other peoples’ reactions. As a result, individuals often fail to observe that other people are responding to them in a broadly benign manner. Another consequence of the shift to an internal focus of attention is an increased awareness of feared sensations.

Second, the model proposes that individuals use internally generated information to create an impression of how they appear to other people. The information drawn upon includes feelings of anxiety and negative self-imagery. Individuals will often overestimate how anxious they look, because they are assuming that they look as anxious as they feel (for example, ‘I feel hot therefore I must be really red in the face’). Negative images are common. Images usually come to mind from an observer perspective rather than a personal (or field) viewpoint, and so it is natural that the images are assumed to be an accurate representation of how the individual looks to other people.

Third, the use of safety behaviours, which are motivated by the desire to prevent or minimise the consequences of feared outcomes (such as sounding stupid or blushing), further maintains social anxiety and negative social beliefs. Common safety behaviours in social anxiety include avoiding eye contact, preparing topics of conversation in advance, wearing lots of make-up, and agreeing with others. Safety behaviours are unhelpful for a number of reasons. They prevent the individual from discovering that the feared outcome was very unlikely to happen anyway. For example, ‘the only reason no-one spotted me blushing was because I was wearing thick foundation’ (rather than because it was not particularly obvious to others anyway). Safety behaviours can heighten self-focus and monitoring, as an individual checks that the safety behaviours are ‘working’. Safety behaviours can directly cause feared symptoms. For example, covering your cheeks to prevent blushing can make you hotter and cause flushing. Safety behaviours can make one appear withdrawn and unfriendly. Behaviours such as avoiding eye contact or keeping conversations short can contaminate the social interaction and give the impression that one is not interested. Finally, safety behaviours can draw attention to feared behaviours. For example, speaking very quietly may cause others to lean in and pay especially close attention in order to hear what is said.

Safety behaviours comprise a broad range of overt behaviours and mental operations. Some safety behaviours involve avoidance, such as speaking less and avoiding eye contact, whilst others are concerned with making a good impression, for example checking you are coming across well and preparing topics in advance. Whilst it is suggested that both groups of safety behaviours are unhelpful as they prevent disconfirmation of negative beliefs and increase anxiety, only avoidance behaviours contaminate the social situation by making the individual appear withdrawn and unfriendly. Three studies have provided support for the distinction between avoidance and impression management safety behaviours. Plasencia et al. (2011) conducted a factor analysis of data from the Social Behaviour Questionnaire which confirmed the existence of the two factors. In addition, correlational analyses indicated that both sets of safety behaviour appear to maintain social anxiety, but only the avoidance behaviours had a negative effect on other people. In an earlier study, Hirsch et al. (2004) examined correlations between subsets of items of the Social Behaviour Questionnaire and a questionnaire measuring the quality of a conversation as rated by the conversation partner. They found that items assessing avoidance behaviours were significantly correlated with negative ratings of the conversation, whilst items assessing impression management behaviours were not. Extending these correlational studies, a recent experimental study (Gray and Clark submitted) directly manipulated the use of safety behaviours during a conversation task. The pattern of results was as expected, with use of both safety behaviour types increasing anxiety, but only avoidance behaviours resulting in a negative response from the conversation partner.

Further unhelpful processes include anticipatory worry and post-event processing. Before a social event, individuals with social anxiety will review what they think is going to happen in detail. Negative predictions will prevail and are associated with anxiety and a host of memories of past failures and negative self-images. This worry is often enough to stop someone entering a social situation in the first place. If they do manage to go, they will be cued up to interpret social failings. Despite some brief relief on leaving a social situation, socially anxious individuals often describe a continued cycle of negative thoughts and distress. Due to the inherently ambiguous nature of most social situations, it is rare that people receive an unquestionable seal of social approval. This ambiguity will usually equate to doubt for the socially anxious individual and in turn initiates a ‘post-mortem’. Post-mortems involve detailed revisiting of the previous event. However, because attention is trained internally during social events, and the focus is on negative thoughts, feelings and images, it is this that is reviewed in detail (especially the most distressing moments), rather than the objective facts of the event. As a result, the event will most likely be labelled a failure. Intense humiliation and shame commonly run alongside these ruminative thoughts. The post-mortem process can continue for days and sometimes weeks after an event.

We suggest that the cognitive model of Clark and Wells (1995) has the potential to be a good fit for an adolescent population. For example, self-focused attention is emphasised in this model and it is a construct that has clear parallels with self-consciousness (Stein 2015) which is heightened during adolescence. Likewise, the concept of safety behaviours, which is emphasised in the model, may be pertinent to a teenage population. Avoidant safety behaviours may elicit particularly negative responses amongst adolescent peers, who as a group are especially sensitive to perceived peer rejection compared to children and adults (see Kilford et al. (2016) for a review). The rest of this review article is therefore concerned with two main questions. First, what evidence is there to support the application of the cognitive model of Clark and Wells (1995) to adolescents? And second, what are the developmentally specific processes that need to be considered for the successful application of the model? We note that quite a few of the maintenance processes that we discuss are highlighted in other prominent models, particularly the model of Rapee and Heimberg (1997), but those models also emphasise some other processes that are not included in this review.

## A Review of Studies Examining the Applicability of the Cognitive Model to Adolescents

### Methodology

We describe a review undertaken to assess the available evidence in relation to the cognitive model of Clark and Wells (1995) in adolescents. The studies we are interested in focus on the *maintenance* of social anxiety rather than the development or aetiology of the disorder. That is not to say that it is not valuable and important to understand the genesis of social anxiety disorder in young people. Rather, we are concerned here in delineating the mechanisms by which social anxiety is maintained in adolescents as a route to developing targeted and effective therapeutic interventions. Studies including young people aged 11–18 years are included. This age range was selected for two reasons. First, we are interested in the adolescent period because this is a peak period of onset of SAD. Second, whilst a definition of adolescence as defined by years is arbitrary because the on- and off-set of puberty will vary from individual to individual, we selected the upper and lower limits of 11 and 18 years as these coincide with the start and end of secondary school in many countries. Studies with a lower age limit below 11 years were retained if the average age was at least 12 years. We included data on non-clinical as well as clinical populations. As noted by Stopa and Clark (2001), social anxiety is continuously distributed in the general population and so comparing (non-clinical) individuals at the relatively high and low ends of a measure of social anxiety seems a meaningful way of researching psychological processes implicated in social anxiety.

**Fig. 2:**
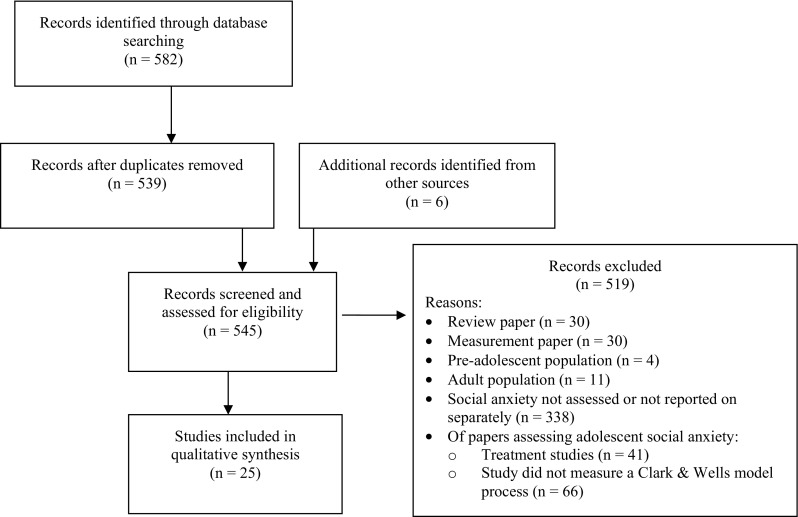
Flow diagram showing selection of papers

We searched the databases PsycINFO and WoK for peer-reviewed articles written in English published between 1995, and the date the search was conducted (28 November 2017). The following search terms were used: (social* AND (anxiet* OR anxious* OR phobi*)) AND ((cognitive AND model) OR (wells AND clark) OR (cognit* OR assumption* OR (interpretat* AND bias) OR belief*) OR image* OR (safety AND behav*) OR (self AND focus* AND attention*) OR ((event AND processing) OR worry OR worri* OR ruminat*)) AND (teen* OR adolescen* OR young OR youth*). To be included studies had to include adolescent samples in which social anxiety symptoms had been assessed as well as at least one of the psychological variables specified in the cognitive model of Clark and Wells (1995). Review papers, studies evaluating measures and scales, and treatment studies were excluded. A PRISMA flowchart showing the selection of papers is presented in Fig. 2. Twenty-five studies are included in the final review (please see Table 2 for a full list of included studies).

**Table 2:** Studies included in review

Author	Year	*N*	Age range (y)	Mean age (SD)	Recruitment	Social anxiety measure
Alfano, Beidel and Turner	2006	48	12–16	13.6 (1.28)	Clinic sample recruited from anxiety outpatient clinic; healthy controls recruited via advert	SPAI-C
Alfano, Beidel and Turner	2008	63	Not reported	13.54 (1.21)	Clinic sample recruited from anxiety outpatient clinic; healthy controls recruited via advert	SPAI-C
Anderson and Hope	2009	392	13–17	14.50 (1.27)	Community sample recruited from school	SPAI-C
Anderson, Veed, Inderbitzen-Nolan and Hansen	2010	170	13–17	14.7 (sd not reported)	Community sample recruited from school	SAS-A, SPAI-C
Blöte, Miers, Heyne, Clark and Westenberg	2014	161	14–18	16.00 (1.38)	Community sample recruited from school	SAS-A
Erath, Flanagan and Bierman	2007	84	11–13	Not reported	Community sample recruited from school	SAS-A
Giannini and Loscalzo	2016	65	14–17	15.43 (1.00)	Community sample recruited from school	SPIN
Haller, Doherty, Duta, Kadosh, Lau and Scerif	2017	51	14–19	16.73 (1.26)	Community sample recruited from school	SAS-A
Haller, Raeder, Scerif, Kadosh and Lau	2016	95	14–17	16.67 (1.05)	Community sample recruited from school	SAS-A
Hignett and Cartwright-Hatton	2008	124	12–18	12.1 (0.25)	Community sample recruited from school	SPAI-C
Hodson, McManus, Clark and Doll	2008	171	11–14	12.24 (0.97)	Community sample recruited from school	SPAI-C
Loscalzo, Giannini and Miers	2017	90	13–17	15.30 (1.06)	Clinic sample recruited from outpatient clinic; healthy controls recruited from schools	SPIN
Miers, Blote, Bogels and Westenberg	2008	73	11–17	13.61 (36.82)	Community sample recruited from school	SAS-A
Miers, Blöte, Heyne and Westenberg	2014	331	9–17	13.34 (2.25)	Community sample recruited from school	SAS-A
Miers, Blote, Sumter, Kallen and Westenberg	2011a	127	9–17	13.02 (2.20)	Community sample recruited from school	SAS-A
Morgan and Banerjee	2006	56	11–13	12.65 (sd not reported)	Community sample recruited from school	SAS-A
Parr and Cartwright-Hatton	2009	36	13–17	14.74 (1.48)	Community sample recruited from school	SPAI-C
Pergamin-Hight, Bitton, Pine, Fox and Bar-Haim	2016	113	6–18	12.40 (3.16)	Clinic and control samples recruitment method not reported	SPAI
Rabner, Mian, Langer, Comer and Pincus	2017	60	13–18	14.9 (1.6)	Clinic sample recruited from anxiety outpatient clinic	MASC
Ranta, Tuomisto, Kaltiala-Heino, Rantanen and Marttunen	2014	135	15–16	15.9 (0.32)	Community sample recruited from school	SPIN
Rheingold, Herbert and Franklin	2003	66	12–17	15.16 (1.4)	Clinic sample recruited from anxiety outpatient clinic and advert; healthy controls recruited via advert	SPAI-C
Rudy, Davis and Matthews	2014	245	8–16	13.27 (2.14)	Community sample recruited from school	SPAI-C
Schreiber and Steil	2013	62	14–20	16.6 (2.21)	Clinic sample recruited from anxiety outpatient clinic; healthy controls recruited via advert	SPAI
Schreiber, Höfling, Stangier, Bohn and Steil	2012	581	14–19	16.49 (1.67)	Community sample recruited from school	SPAI
Thomas, Daruwala, Goepel and De Los Reyes	2012	40	14–17	15.15 (0.97)	Clinic and control samples recruited via advert	MASC

*SPAI-C* Social Phobia Anxiety Inventory for Children (Beidel et al. 1995), *SAS-A* Social Anxiety Scale for Adolescents (La Greca and Lopez 1998), *SPIN* Social Phobia Inventory (Connor et al. 2000), *SAS-CR* Social Anxiety Scale for Children Revised (La Greca and Stone 1993), *SPAI* Social Phobia Anxiety Inventory (Turner et al. 1989), *MASC* Multidimensional Anxiety Scale for Children (March et al. 1997)

Effect sizes (ES) are provided. These are either reported directly from studies (where provided), or we have calculated these if they were not reported. For studies involving correlational analysis we have reported *r* and for group differences studies we have reported Cohen’s *d. r* is interpreted according to Cohen’s (1988) criterion whereby a small effect is at least 0.1, a medium effect is of a magnitude of at least 0.3, and an effect size greater than 0.5 is deemed large. Cohen’s *d* is interpreted as such: at least 0.2 is a small effect, at least 0.5 is a medium effect, and at least 0.8 is a large effect.

### Negative Cognitions and Perceived Social Danger

#### Negative Social Attitudes and Cognitions

According to the cognitive model, individuals with social phobia hold dysfunctional assumptions about themselves that are activated in anticipation of a social situation (“I must always speak eloquently”). Negative social cognitions about oneself (e.g. “I will stutter”) and about other peoples’ reactions (“people will think I’m stupid”) predominate. Supportive evidence for this hypothesis in adolescents is provided by three questionnaire studies. Schreiber et al. (2012) and Hodson et al. (2008) reported on studies undertaken with German (aged 14–20 years) and UK (aged 11–14 years) school samples respectively. In their large non-clinical sample of 581 adolescents and young adults, Schreiber et al. (2012) found that those scoring in the upper quartile of the German version of a measure of social anxiety, the Social Phobia Anxiety Inventory (SPAI; Turner et al. (1989)) endorsed more frequent negative social cognitions in social situations (e.g. ‘I am boring’, ‘I will blush’) on the Social Cognitions Questionnaire (Clark 2003). In the only study to look at negative social attitudes, the authors also found that those scoring in the top quartile on the SPAI rated themselves more highly on negative social attitudes (e.g. ‘I must be witty and intelligent at all times’) on the Social Attitudes Questionnaire (Clark 2003) compared to those in the lower quartile. Effect sizes were large for both findings; *d* = 1.56 and 1.74, respectively. In regression analyses, both social cognitions and social attitudes were significant independent predictors of social anxiety across the whole sample, but they were also predictors of depression. Similarly, the smaller UK study with 171 non-clinical adolescents (Hodson et al. 2008) found that those scoring in the top quartile on the Social Phobia Anxiety Inventory for Children (SPAI-C; Beidel et al. (1995)) reported more frequent negative social cognitions on the Social Cognitions Questionnaire compared to those in the middle quartiles (*d *= 1.20) and the lowest quartile (*d *= 1.40). The ‘middle’ and ‘low’ groups did not differ from each other (*d *= 0.39). Also in line with the findings of Schreiber and colleagues, regression analyses indicated that social cognitions were a significant independent predictor of both social anxiety and depression. In the third questionnaire study, Rudy et al. (2014) examined self-report questionnaire measures of negative social cognitions, self-efficacy and social anxiety in a US sample of 260 healthy adolescents aged 8–16 years. Negative social cognitions (e.g. ‘I sound stupid’) were associated with social anxiety both directly (*r *= 0.66) and indirectly via their effect on self-efficacy.

Using a semi-structured interview methodology, Ranta et al. (2014) asked adolescents to identify a time when they had felt very socially anxious and to then recall the thoughts that they had during the experience. Those scoring higher on the Social Phobia Inventory [SPIN; Connor et al. (2000)] recalled more negative thoughts than those scoring lower (*d *= 0.48). Although the numbers of young people with clinical levels of SAD were small, the findings were similar when the authors compared those assigned a diagnosis of clinical or subclinical social anxiety disorder (*n* = 17) compared to those with no diagnosis of SAD (*n* = 116; *d* = 0.58). Negative thoughts recalled by all participants were most commonly self-focused rather than focused on other people or the interaction. In another study comparing a sample of adolescents with social anxiety disorder with a group of healthy controls, Alfano et al. (2006) asked participants to engage in a role-play task. The authors found that not only did socially anxious youth make more negative predictions about their performance than controls (*d *= 1.61), but they also went on to believe they performed less well than they had expected, whereas control participants did not draw negative conclusions about their performance (*d* = 1.56). Negative self-talk was significantly more frequent in socially anxious adolescents compared to controls (*d *= 0.69) and compared to socially anxious children (*d *= 0.67).

The studies described all report consistent findings that are supportive of the cognitive model. Negative social cognitions and attitudes are elevated in adolescents endorsing more social anxiety. Two studies have suggested that this holds in clinical samples also. However, there are limitations to the data, most notably, all of the studies are correlational^1^ and so the issue of whether cognitions are causally implicated in social anxiety cannot be resolved.

#### Negative Interpretation Bias

Social events are rarely conclusively negative, and yet socially anxious individuals will often draw very negative conclusions about their social performance and others’ reactions. Considerable evidence has amassed in support of the presence of a bias amongst socially anxious adults towards interpreting ambiguous events in a negative way and appraising mildly negative events catastrophically (Mobini et al. 2013). A study by Stopa and Clark (2000) provides particularly clear evidence for this. Adults with social anxiety disorder and healthy controls were presented with ambiguous scenarios depicting social and non-social events, and with unambiguous scenarios depicting mildly negative social events. Socially anxious adults not only made more negative interpretations of ambiguous social scenarios, but they were also more likely to catastrophize unambiguous, mildly negative social events. In pre-adolescent children there are fewer studies, but a review concluded that there is reasonable evidence for the association of a negative interpretation bias with social anxiety (Halldorsson and Creswell 2017).

Turning now to adolescents, in their review, Haller et al. (2015) note that the refinement of neurocognitive abilities such as perspective-taking coupled with exposure to increasingly large and complex social networks may make some adolescents especially susceptible to drawing negative social conclusions compared to children and adults. Studies examining interpretation bias in adolescent social anxiety have used a range of methods. Four studies, with Dutch (Miers et al. 2008), Italian (Giannini and Loscalzo 2016; Loscalzo et al. 2017) and US (Rheingold et al. 2003) samples, have employed a questionnaire measure of interpretation bias. In three of the studies (Miers et al. 2008; Giannini and Loscalzo 2016; Loscalzo et al. 2017), participants were given the Adolescent Interpretation and Belief Questionnaire (Miers et al. 2008). The questionnaire presents a series of ambiguous social and non-social scenarios followed by three possible interpretations (positive, negative and neutral). Respondents are asked to rate how likely the interpretations are to pop into mind and how much they believe them.

Miers et al. (2008) found that adolescents scoring high (in the top 10%) on a measure of social anxiety (the Social Anxiety Scale for Adolescents [SAS-A; La Greca and Lopez (1998)]) made significantly more negative interpretations of social events than the average anxiety group (those scoring in the 45–55th centile on the SAS-A; *d* = 1.18) and believed these more strongly (*d* = 0.54). There were no differences in positive interpretation of social events (*d* = 0.28). High social anxiety adolescents were also more likely to make negative interpretations (*d *= 0.62) of non-social situations and to believe these negative interpretations (*d *= 0.59) more than average anxiety adolescents. In line with these findings, Giannini and Loscalzo (2016) found non-clinical high scorers (on the SPIN) endorsed more frequent negative interpretations of social situations (*d* = 0.84) and believed these more than average scorers (*d* = 1.19). However, there were some differences between the two studies. First, in this study, high anxiety adolescents also made fewer positive interpretations (*d* = 0.70) than the average anxiety scorers (whereas no differences were found on this measure by Miers et al. (2008)). Second, this study did not find any differences in interpretation of non-social situations, whereas a difference had been identified in the study by Miers et al. (2008).

The study by Loscalzo et al. (2017) compared 50 adolescents with a clinical diagnosis of social phobia with non-clinical adolescents scoring high or low on the SPIN. This study also used the Adolescent Interpretation and Belief Questionnaire. In line with the findings of Miers et al. (2008) there were no group differences in positive interpretations of social situations, but the social anxiety disorder group and the high social anxiety group made more frequent negative interpretations of social situations than those in the low social anxiety group (*d* = 1.82; 1.82, respectively). Those in the social anxiety disorder group believed the negative interpretations more than those in the high (*d *= 0.56) and low (*d* = 0.75) social anxiety groups, who did not differ from each other (*d *= 0.14). In terms of content-specificity, adolescents with social anxiety disorder also made more negative interpretations of non-social ambiguous scenarios and believed these more strongly than non-clinical adolescents with high or low social anxiety. Another correlational study was undertaken by Rheingold et al. (2003). Adolescents with a diagnosis of social anxiety disorder and healthy controls completed a questionnaire measure assessing judgments of the likelihood and cost of social and non-social events. Socially anxious youth overestimated the cost (*d *= 1.87) and probability (*d *= 1.50) of negative social events and the cost of negative non-social events (*d *= 0.84) compared to controls, even after controlling for depression symptoms. However, none of these four studies assessed interpretations ‘on-line’ and so we cannot determine whether they are measuring direct interpretations of events as they occur or rather some pre-existing negative beliefs.

Addressing the limitations associated with this method, a recent study by Haller et al. (2016) used a novel picture-based paradigm to measure interpretation bias amongst 95 school-based non-clinical 14–17-year olds. Participants were shown an ambiguous social scene with a photograph of themselves inserted as the protagonist. They were presented with neutral, negative and positive interpretations. Social anxiety was significantly negatively correlated with positive interpretation ratings (*r* = − 0.48) and significantly positively correlated with negative interpretation ratings (*r* = 0.45). Extending this finding, Haller et al. (2017) asked a community sample of adolescents to interpret ambiguous social situations presented with naturalistic photographs. Eye tracking data were gathered as a proxy measure of attentional allocation. Social anxiety levels predicted a tendency to make more negative and less positive interpretations. Furthermore, participants who spent more time on facial displays made more threatening interpretations. In a study measuring online interpretation biases in a sample of young adolescents with social anxiety disorder and a group of healthy controls (Pergamin-Hight et al. 2016) found that the clinical group made more negative interpretations compared to the control group (*d *= 0.47).

Seven studies have examined interpretation biases in social anxiety in adolescents. All point to an association between social anxiety and an increased tendency to draw negative interpretations of ambiguous social scenarios and to believe these more strongly. Findings are more mixed about the association between social anxiety and positive interpretations of social scenarios, and in relation to the interpretation of non-social events. In addition, as yet, no studies have examined whether socially anxious individuals tend to catastrophize in response to mildly negative social scenarios as would be predicted by the model.

### Processing of the Self as a Social Object

#### Enhanced Self-Focused Attention Linked to Reduced Processing of External Social Cues

The model would predict that when a social threat is perceived, socially anxious individuals shift their attention internally and reduce processing of external social cues. Indeed, socially anxious adolescents very often talk about being painfully self-conscious in social situations. To test this hypothesis, the questionnaire-based study of Hodson et al. (2008) included the Focus of Attention Questionnaire (FAQ; Woody (1996). High socially anxious adolescents reported higher levels of self-focus compared to middle (*d* = 0.49) and low socially anxious youth (*d* = 1.09). Self-focused attention was associated with social anxiety across the whole sample (*r *= 0.42), and it was also an additional independent predictor of social anxiety, with and without simultaneous adjustment for depression scores. Consistent with this finding, Schreiber et al. (2012) also found that high socially anxious adolescents reported higher levels of self-focused attention (measured by the FAQ) compared to low scorers (*d* = 1.15). Self-focused attention was not found to be a significant independent predictor of social anxiety across the whole sample. The authors also looked at external focus of attention, measured by certain items of the Focus of Attention Questionnaire. No differences between groups were found on this subscale. The authors suggested this null finding might have resulted from the lack of specificity regarding the type of external focus captured by the items.

A more ecologically valid study was undertaken by Blöte et al. (2014). One hundred and sixty-one non-clinical 14–18-year olds gave a speech to a pre-recorded neutral audience. Questionnaire measures of social anxiety and self-focus (using the FAQ), performance expectation and audience perception were completed. In relation to self-focused attention, it was found that as expected social anxiety was associated with higher self-focus (*r *= 0.43). The relationship between social anxiety and audience perception was found to be partially mediated by negative expectations of performance and self-focused attention. In line with the findings of Schreiber et al. (2012), external focused attention was not correlated with social anxiety (*r *= 0.12).

A longitudinal study was undertaken with a sample of unselected Dutch youth to examine pathways of social avoidance through adolescence and whether social anxiety and psychological processes, including self-focused attention (using the FAQ), discriminated between the pathways (Miers et al. 2014). A group of adolescents showed increasing social avoidance through adolescence, and another group showed consistently low avoidance. Self-focused attention was not found to discriminate between the two groups. However, the study was designed to identify vulnerability factors for the development of social avoidance rather than current maintenance factors.

A number of studies have been reported on that have examined the role of self-focused attention in social anxiety. In all but one study (Miers et al. 2014), self-focused attention was found to be related to social anxiety (or group differences were shown) with a medium effect. However, conclusions are limited by a number of issues. First, none of the studies were undertaken with clinical populations. Second, all of the studies used the FAQ to measure self-focused attention and often only used a small number of items taken from the questionnaire. Although the measure has been found to have moderate internal consistency, full psychometrics have not been reported with adolescents. Third, the studies cannot illuminate us on the causal role of self-focused attention in social anxiety; for this, experimental studies are needed.

#### Negative Observer-Perspective Social Images

The majority of adults with SAD report experiencing negative observer-perspective self-images in social situations (Hackmann et al. 2000). These are often related to events dating back to around the onset of the disorder. A number of studies have examined negative self-imagery and social anxiety in adolescents. Three of these used questionnaire or interview methods. Ranta et al. (2014) found that high socially anxious adolescents (compared to low scorers) and adolescents with SAD or subclinical SAD (compared to no diagnosis) report more negative observer-perspective self-images (*d *= 0.45 and *r *= 0.47, respectively). Similarly, in their study with unselected adolescents, Schreiber et al. (2012) found that high social anxiety adolescents reported more frequent negative self-images than low scorers (*d* = 0.81). Frequency of negative self-images was also found to independently predict social anxiety in the group as a whole. The study used the Questionnaire of Recurrent Images in Social Phobia (QRI-SP; Schreiber et al. (2009)) which is based on the semi-structured developed by Hackmann et al. (2000) for adults to assess negative self-imagery. However, the study only used a single item of the measure and this may not be reliable with a multi-faceted construct such as self-imagery. Addressing this limitation, Schreiber and Steil (2013) administered the full QRI-SP as a semi-structured interview to 31 adolescents with a clinical diagnosis of SAD and to a matched sample of 31 healthy adolescents. They found that whilst all adolescents had experienced negative self-imagery in social situations in the past, those with SAD experienced images more frequently (*d *= 0.84), and they were more distressing (*d *= 0.80) and more vivid (*d *= 0.54). These images were more likely to be experienced from the observer perspective by those with SAD compared to controls (*d *= 0.75). Almost half of controls (45.2%) and two-thirds of the SAD group (64.5%) identified a socially traumatic event linked to the negative self-image, but this proportion did not differ between the groups. A study by Hignett and Cartwright-Hatton (2008) further examined the hypothesis that the negative images experienced by socially anxious individuals tend to be from the observer perspective. One hundred and twenty-four unselected adolescents aged 12–18 years gave a brief talk to a camera. They were then asked to bring to mind how they thought they had appeared and rate the extent to which this image was from the field or observer perspective. A modest but significant association between social anxiety (SPAI-C) and tendency to take the observer perspective (*r *= 0.20) was found.

A convincing demonstration of the causal role of negative self-imagery in social anxiety in *adults* was provided by Hirsch et al. (2004) (see also Hirsch et al. (2003)). In their study, socially anxious adults took part in two conversations, holding either a negative or a benign image in mind. Findings were in line with the cognitive model: compared to a benign image, holding a negative social image in mind increased anxiety, negative appraisals and use of safety behaviours and led to a poorer judgement by the conversation partner. Alfano et al. (2008) undertook a similar experimental study with 63 SAD and healthy control adolescents. Adolescents undertook videotaped role-play and read aloud tasks. Afterwards, they rated their anxiety and their social performance and an independent assessor made objective ratings of their performance. Half the control participants were instructed to engage in negative self-imagery during the tasks and the other half received no instructions. Contrary to the hypotheses and the findings of Hirsch et al. (2004) with adults, few differences were found in observer- or self-rated anxiety or performance between the two control groups. The SAD group was consistently rated as more anxious and less socially competent. The authors conclude that negative self-imagery may be a consequence of social anxiety rather than a causal factor as suggested by the cognitive model. However, it seems plausible that the null finding was due to design issues, in the sense that the manipulation of imagery was between-subjects which would give lower statistical power and also the control condition was not clearly defined. It is difficult to know what the imagery group was being compared to because participants in the comparison groups did not receive any instructions and were not subsequently asked about thought content during the experimental procedure. In comparison, the study of Hirsch et al. (2004) included a controlled within-subjects comparison (benign imagery). It therefore seems that there is scope for further causal experiments in adolescents including a tighter control over the experimental manipulation.

Video feedback with careful verbal preparation beforehand is a core cognitive therapy technique aimed at correcting negative and distorted self-imagery. Studies with adults have shown that the technique leads to more accurate appraisals of performance and reduced anxiety (Warnock-Parkes et al. 2017), providing corollary evidence for the role of self-images in social anxiety. Turning to studies with adolescents, Parr and Cartwright-Hatton (2009) examined the effect of video feedback with 36 highly socially anxious 14–17-year olds. Video feedback was provided with careful preparation to prepare an unbiased mode of processing (c.f. Warnock-Parkes et al. (2017)). Compared to control participants (who sat quietly), young people who received video feedback after giving a speech felt less anxious about giving a subsequent speech (*d *= 1.43), predicted that they would perform better (*d *= 1.16) and went on to rate the later speech as better (*d *= 1.22). A somewhat similar study was undertaken with 11–13-year olds, scoring high and low on the SAS-A (Morgan and Banerjee 2006). Adolescents took part in a role-play task, with half receiving video feedback and half completing a distractor task prior to rating their performance. In contrast to the findings of Parr and Cartwright-Hatton (2009), the study did not find any improvements in participants’ performance ratings after video feedback for the high (*d *= 0.17) or low anxiety groups (*d* = 0.16). However, participants were not given any preparation for the video feedback. We know from adult research that whilst almost all studies have found the technique to be helpful, the two studies that have failed to find positive effects of video feedback (Rodebaugh 2004; Smits et al. 2006) did not include verbal preparation before watching the video. Careful verbal preparation is needed in order to overcome the processing biases that can undermine the effectiveness of the technique.

Reviewing the studies that have looked at self-imagery in adolescent social anxiety we find consistent results from the three questionnaire and interview studies (Ranta et al. 2014; Schreiber et al. 2012; Schreiber and Steil 2013), two of which reported on clinical samples. With a moderate effect, socially anxious youth reported more frequent negative observer-perspective social images compared to low scorers. The only experimental study to be undertaken (Alfano et al. 2008) did not find a detrimental effect of asking young people to engage in negative self-imagery, but this may be due to design issues. Looking at the relevant data on video feedback, we find that in line with adult reports, the study that undertook video feedback with careful verbal preparation yielded positive effects of the technique, whilst the study that did not include it failed to find a benefit. Experimental studies with clinical and non-clinical samples including valid manipulations are still needed to test the model’s hypothesis that negative self-imagery plays a causal role in adolescent social anxiety.

#### Use of Internal Information

The cognitive model would predict that socially anxious individuals use internal information made accessible by self-focused attention to make excessively negative inferences about how they look to others. This hypothesis has been supported in studies with adults. For example, Mansell and Clark (1999) asked adults with high and low levels of social anxiety to undertake a speech task. Participants rated their perceived body sensations and how they thought they appeared during the task. An independent assessor also rated how they came across. Amongst adults with high, but not with low social anxiety, a significant correlation was found between perceived body sensations and how anxious they thought they looked. The correlation between assessor ratings and perceived body sensations was non-significant for both groups. The findings are consistent with the suggestion that socially anxious individuals use perceived bodily sensations to make erroneous negative judgements about how they appear.

No studies have been undertaken examining this hypothesis with adolescents. Ancillary support comes from three studies that have compared subjective and objective measures of arousal in adolescents. In a study by Anderson et al. (2010), subjective physiological arousal and heart rate during a speech task were compared between 170, 13–17-year olds with SAD, high social anxiety adolescents and controls. SAD and high social anxiety adolescents both endorsed elevated self-reported arousal (measured on a subscale of items from the on Beck Anxiety Inventory (BAI) (Beck et al. 1988) compared to controls (SAD vs. low *d* = 0.71; high social anxiety vs. low *d *= 0.45), but did not differ from one another (*d* = 0.33). There were no differences in heart rate between the groups. Comparable findings were reported in an earlier study by Anderson and Hope (2009), comparing SAD (*n* = 85) with controls (*n* = 285) during a speech and a conversation task. There was no significant difference between groups in heart rate reactivity during the speech (*d *= 0.17) or the conversation (*d *= 0.15). However, adolescents with SAD rated themselves as more physiologically aroused on the BAI during both the speech (*d *= 0.81) and the conversation task (*d *= 0.65). In a non-clinical sample of 136, 9–17-year olds, high and low socially anxious youth were compared on self-reported and objective measures of heart rate and sweating during a speech task (Miers et al. 2011a). High social anxiety participants reported a higher heart rate and sweatier palms (*d* = 0.52), but no differences in heart rate or skin conductance levels were found.

These three studies are consistent in their finding that whilst socially anxious adolescents and controls are comparable on indices of objective arousal, the socially anxious groups consistently overestimate their bodily symptoms of anxiety (Siess et al. 2014). It could be argued that these results are in line with the cognitive model because when socially anxious adolescents overestimate their physical sensations this may lead them to overestimate how anxious they *look*. However, studies in which adolescents are also asked how they think they appear are needed in order to directly test the hypothesis.

### Use of Safety Behaviours

In adults, studies have found that socially anxious adults use safety behaviours in social situations more than those who are not socially anxious (e.g. Cuming et al. (2009), Pinto-Gouveia et al. (2003)). We also have robust experimental data pointing to the causal role that these safety behaviours play in social anxiety in adults (e.g. McManus et al. 2009, 2008). The data in relation to safety behaviours in children are scant (Halldorsson and Creswell 2017), but four studies have addressed the question in adolescents and they find similar results to in adults.

In the questionnaire study of Hodson et al. (2008), adolescents completed a self-report measure of safety behaviour use, the Safety Behaviour Questionnaire (Clark 2003). High social anxiety adolescents endorsed a greater use of safety behaviours compared to middle (*d* = 0.65) and low (*d* = 1.01) scorers, who did not differ from each other (*d* = 0.32). Safety behaviour use was significantly associated with social anxiety across the whole sample (*d *= 0.94), but it did not emerge as a unique predictor of social anxiety. In line with this study, Schreiber et al. (2012) using the same measure of safety behaviours (translated into German) found that high socially anxious 14–20-year olds used safety behaviours more than low socially anxious youth (*d *= 1.34). In contrast to the study of Hodson et al. (2008), they did find that across the whole sample safety behaviour use was a significant independent predictor of social anxiety but not of depression. Ranta et al. (2014) asked unselected adolescents about the safety behaviours they had used when they had felt socially anxious. 17% of the whole sample reported using at least one safety behaviour when they felt socially anxious. Safety behaviours were more frequent in the high social anxiety group compared to the low group (*d* = 0.68), and amongst those with a clinical or subclinical diagnosis of SAD compared to without (*r* = 0.63). The fourth study was undertaken by Thomas et al. (2012). The authors administered a measure of safety behaviours developed with adults, the SAFE (Cuming et al. 2009), and a measure of social anxiety to a group of adolescents referred to a clinic with possible social anxiety and to a group of community controls. In line with hypotheses, social anxiety was positively correlated with safety behaviour use across the whole sample (*r* = 0.49), and the socially anxious group endorsed significantly greater safety behaviour use than the community controls (*d* = 0.77).

In summary, four studies have looked at safety behaviours in adolescent social anxiety. A medium to large effect was found. Unfortunately, all of the studies were correlational. Studies modulating the use of safety behaviours and examining the effect (on, for example, anxiety, cognitions, self-focus and social performance) will be important in order to test the hypothesis that these behaviours are causally implicated in social anxiety. Due to the sensitised peer environment during adolescence we would expect avoidance safety behaviours to result in particularly negative reactions from their peers, and so socially anxious adolescents will be susceptible to becoming locked into a vicious cycle and subjected to peer victimisation, which we know is especially common in adolescents (Troop-Gordon 2017). Experimental studies will be valuable here. Furthermore, there are as yet no studies examining the putative sub-types of safety behaviours, their effects amongst adolescents or developmental influences on the use of safety behaviours. For example, it is conceivable that there may be a developmental progression in young people’s use of safety behaviours. As children move into and through adolescence they may become increasingly sophisticated in their use of safety behaviours, for example moving from a reliance on avoidance behaviours towards increasing adoption of impression management behaviours.

The notion of safety behaviours provides a different perspective on the understanding of the inhibited or withdrawn behaviours of people who are socially anxious. Traditionally, these behaviours have been interpreted as a sign that the individual lacks social skills (Wong and Rapee 2015). However, treated adults do not show ongoing social skills deficits (for a review see Hofmann (2007)). When individuals are not anxious they do not show deficits in social skills. Any deficits in performance seem to be largely restricted to situations in which they are anxious, which suggests that they are an anxiety response rather than an indication of a lack of knowledge or ability (Alden and Taylor 2004). It seems likely that the apparent social skills deficits are in fact the observable safety behaviours (and the avoidance behaviours in particular).

Turning to studies with young people, in pre-adolescents a recent review concluded that there is evidence for an association between social anxiety disorder and a skills deficit (Halldorsson and Creswell 2017). In adolescents, a number of studies have found that peers rate socially anxious youth as less socially skilled than non-anxious peers (e.g. Miers et al. 2011b, 2010). However, as highlighted by Wong and Rapee (2015), it is not possible to determine the direction of causality from these studies. Further, none of these studies have considered the contaminating effect of safety behaviours on social skills. As such, it is difficult to draw conclusions from the available studies about whether there is a latent skills deficit. It will be important to test whether in adolescents, as in adults, the apparent performance deficits can in fact be explained by observable safety behaviours. Indeed, it seems plausible that if, as we suggest, socially anxious younger people do rely on avoidance safety behaviours more than impression management strategies, they may present as even more withdrawn and inhibited than socially anxious adults. As such, a performance deficit account could be drawn upon more readily with this population, despite the absence of an extant deficit. It will be important to test this hypothesis, as it has implications for our understanding of the maintenance of social anxiety and for treatment.

### Pre- and Post-event Processing

#### Pre-event Processing

Worry is a feature of all anxiety disorders, and the cognitive model predicts that it is an important maintenance process in social anxiety as well. Examining this hypothesis, the questionnaire studies of Hodson et al. (2008) and Schreiber et al. (2012) both used a single item from the Social Phobia Weekly Summary Scale (Clark 2003) to measure pre-event processing (“over the past week, how often have you gone over in your mind things that you think might go wrong in a social situation before entering the situation”). In the study by Hodson et al. (2008), high socially anxious youth engaged in worry before social situations more than middle (*d* = 0.86) and low (*d* = 0.86) social anxiety groups, who did not differ from each other (*d* = 0.18). Across the whole sample, pre-event processing was significantly associated with social anxiety (*r* = 0.44) but also to depression (*r* = 0.42). It was a significant independent predictor of depression but not of social anxiety. This suggests that the process may not be specific to social anxiety, which is unsurprising given that repetitive thinking processes are implicated in a range of common mental health disorders (Watkins 2008). Schreiber et al. (2012) also found that high socially anxious youth reported more anticipatory worry than low scorers (*d *= 0.84). Again consistent with the findings of Hodson et al. (2008), pre-event processing was found to be an independent predictor of depression but not social anxiety in regression analyses. A correlational study of 60 adolescents with an anxiety disorder diagnosis (Rabner et al. 2017) found a significant correlation between self-reported social anxiety symptoms and worry (*r *= 0.432), as measured by the Penn State Worry Questionnaire (Chorpita et al. 1997).

Studies examining the related idea of anticipated performance criticism are relevant here. A recent study of anticipated audience criticism (Ranta et al. 2014) tested the hypothesis that socially anxious adolescents tend to expect more negative audience reactions in non-threatening situations compared to their peers. Amongst 333 adolescents scoring in the top and bottom quartile of the SAS-A, their hypothesis was confirmed: socially anxious youth demonstrated a tendency to expect negative classmate reactions (measured by self-report questionnaire) when they were the presenter (*r *= 0.60). There were no differences in expectations between anxiety groups when participants were asked to imagine an anxious peer rather than themselves, or in positive expectations of reactions. Similarly, in a study by Blöte et al. (2014), social anxiety was associated with more negative expectations of performance before a speech task (*r *= 0.32). Likewise, asked to rate expectations of their performance in a videotaped role-play task, high socially anxious young adolescents thought they would perform worse than low scorers (*d *= 0.51; Morgan and Banerjee (2006)). Erath et al. (2007) asked 42 high socially anxious and 42 average anxiety adolescents to undertake a videotaped conversation task with a young adult. Higher social anxiety was significantly correlated with expectations of poorer social performance (*r* = 0.27). In addition, negative social performance expectations predicted skill deficits in the conversation task (*r *= 0.25).

These findings are all consistent with the cognitive model; pre-event processing involves a focus on potential negative outcomes of a social situation and an anticipation of social failure. These negative expectations will increase the likelihood that the situation is interpreted negatively, ones focus of attention shifts internally and safety behaviours are utilised, thereby maintaining anxiety. However, although using a variety of methods, all of these were correlational studies with analogue samples.

#### Post-event Processing

Rumination is in many respects a similar process to worry, and like worry it is implicated in a wide range of mental health difficulties (Watkins 2008) including social anxiety. Only two studies have examined this process in adolescent social anxiety. Using comparable methodologies, both Hodson et al. (2008) and Schreiber et al. (2012) examined whether group differences in post-event processing could be identified amongst a stratified non-clinical sample. Both used the Post-Event Processing Questionnaire (Rachman et al. 2000). It comprises 13 items related to how much the individual went over an event afterwards. Hodson et al. (2008) found that high socially anxious youth reported more post-event processing than middle (*d* = 0.62) or low (*d* = 0.95) groups (who did not differ from each other (*d *= 0.30). Post-event processing was significantly associated with social anxiety across the whole sample (*r* = 0.40) and with depression (*r* = 0.28). It was also a significant independent predictor of social anxiety. In the study of Schreiber et al. (2012), high socially anxious youth endorsed more post-event processing than low scorers (*d *= 0.94). In regression analysis, post-event processing was not a significant independent predictor of social anxiety, but it was of depression.

In summary, only two studies have looked at the role of post-event processing in adolescent social anxiety. Whilst both indicate that the process is elevated in adolescents with higher levels of social anxiety (with a medium effect), they were questionnaire studies with analogue samples, limiting the conclusions that we can draw.

### Summary

Reviewing the studies undertaken with adolescent samples we see that there is encouraging support for the hypotheses derived from the cognitive model of Clark and Wells (1995). However, as yet the majority of studies have been undertaken with analogue samples, typically comparing extreme scorers on a measure of social anxiety. This is generally considered to be a valid research strategy given that social anxiety is thought to vary continuously across the population (Stopa and Clark 2001), but it will be important to replicate the findings with adolescent clinical samples. Another limitation of the studies is that the majority of them have been correlational. As such the resultant findings provide promising support for the hypotheses but cannot demonstrate the causal status of the processes in the model. To address this gap in the literature, further experimental studies are needed in which the psychological processes of interest are manipulated and their effect on social anxiety observed. As well as this, when considering the downward application of a static adult model to a dynamic adolescent period, there is a need to consider the developmental influences that have a bearing on these processes. Notwithstanding these caveats, the literature is sufficiently encouraging to suggest that adapting the treatment derived from the model for adolescents might be a promising approach. When considering this, attention must turn to the additional developmentally sensitive elements that may need to be included to explain the maintenance of social anxiety during the teenage years. There are features that are particular to adolescence that may well lock young people into the negative cycles that maintain social anxiety.

## Developmentally Sensitive Factors Relevant to the Application of the Model to Adolescents

As outlined earlier, adolescence is a developmental period associated with particular cognitive, social and familial changes and these may well contribute to the persistence of social anxiety. We will focus here on the two factors that seem most salient and have been subject to the most scientific interest: parental factors and friendships and peer victimisation. Our ambition here is not to undertake a comprehensive review of these factors, but rather to consider whether these factors are associated with social anxiety, and if so, how they may maintain key processes in the cognitive model. We also briefly touch on the relationship between social anxiety and social media use. Although the research field is still relatively small it is an area of particular relevance in relation to adolescents and social anxiety.

### Parenting Factors

Family processes, and parenting processes in particular, are commonly agreed to be a contributory factor in the development and maintenance of child anxiety (Rapee et al. 2009). The vast majority of the research in this area has been conducted with pre-adolescent children (Kendall and Ollendick 2004). However, given that the demands of parenting will shift and change considerably as children move into adolescence, it seems reasonable to think that parental influences on youth anxiety may also change during this time. For example, with increasing independence and autonomy the association between parental factors and child anxiety may be hypothesised to decrease over time. But it is equally conceivable that given the potential importance of parents in helping young people navigate their increasing autonomy, parental factors may be relevant in adolescent anxiety.

The most well-researched dimension of parenting in the aetiology and maintenance of youth anxiety is parental over-control or overprotection. This is defined as a pattern of behaviour involving overly protective, directive and controlling behaviours, even when the situation does not require it, and discouragement of independent problem solving. A recent review concluded that the majority of available studies (75%) point to a significant contribution of parental over-control to adolescent anxiety in general (Waite et al. 2014) and there is some evidence of its association with social anxiety symptoms specifically in pre-adolescents (Halldorsson and Creswell 2017).

In adolescents, three studies are particularly relevant when examining the relationship between parenting and adolescent social anxiety specifically (Loukas 2009; Fisak and Mann 2010; Caster et al. 1999). All were questionnaire studies undertaken with analogue adolescent samples in the USA. Adolescents completed measures assessing social anxiety (the SAS-A) and perceptions of parenting. The study by Loukas (2009) looked at the relationship between social anxiety and the perception of maternal psychological control amongst 479, 10–14-year olds. No significant association was found (*r* = 0.02 for females, and *r *= 0.10 for males). The study by Fisak and Mann (2010) focused on 348 older adolescents (aged 15–18 years). Participants were split into ‘High’ (> 50 on the SAS-A) and ‘Low’ social anxiety groups. The high anxiety group rated their parents as more likely to model social fears, discomfort and avoidance (*d *= 0.39) and to communicate shame and criticism of adolescent’s social interactions and skills (*d* = 0.47). Adolescent perceived parental sociability and tendency to engage in social situations outside the family did not differ between groups (*d* = 0.08). Caster et al. (1999) undertook a large study comparing perceptions of parenting and the family environment made by adolescents categorised as high or low socially anxious. Adolescents were categorised in the high group if they scored at least one standard deviation above their gender and grade average on one or more of a number of measures of social anxiety (including the SAS-A). Those scoring at or below their gender and grade mean were classified as low social anxiety. High socially anxious youth rated all dimensions of the Family Environment Questionnaire (Caster et al. 1999) higher than low socially anxious youth. Specifically, they perceived their fathers and mothers as being more socially isolating, as being more concerned about others’ opinions, more ashamed of their shyness and poor performance, and less socially active (all *d*’s between 0.37 and 0.69). A particularly interesting aspect of this study was the inclusion of parental reports of the family environment (using the same Family Environment Questionnaire). No significant differences in parent ratings of the family environment were found between parents of high and low socially anxious youths.

Overall, two of the three studies have found significant group differences between non-clinical groups scoring high and low on a measure of social anxiety. Interestingly, in one study this did not tally with findings on parent ratings of the family environment. All the studies used measures of perceptions of parenting. This makes sense in many ways, but if anxious adolescents are negatively biased in how they process information then higher scores on negative parenting dimensions may reflect a more general negative bias rather than a specific appraisal of parenting quality. Studies including measures of parenting completed by socially anxious youth and their parents as well as observational assessment of parenting would greatly add to the field. There has also been a notable lack of experimental studies examining the effect of manipulating parent–child interactions. One exception was an elegant study carried out by de Wilde and Rapee (2008) (although with pre-adolescents (mean age 10.19y), hence not reported in detail here). In brief, mothers were either required to be minimally or overly controlling with their children during preparation for a speech task. In a subsequent speech that the children prepared for alone, those whose mothers were overly controlling reported more anxiety than those whose mothers had been minimally controlling. Studies such as this would be especially informative about the relevance of parenting processes in adolescent social anxiety.

We can now consider the ways in which aspects of parenting may relate to the processes specified in the cognitive model. It seems likely that the relationship between parent and child anxiety, beliefs and behaviour is iterative and interactive (Rubin et al. 2009). Parents will bring their own attitudes and assumptions to their parenting practices. These parental beliefs are likely to motivate particular parenting behaviours (Rubin et al. 2009). Parenting beliefs characterised by a heightened perception of threat in the social environment and/or appraisals relating to their child’s vulnerability will most likely lead to anxiety about a child’s ability to thrive in a social environment. As such parents may engage in overprotective or over-controlling behaviours to mitigate their concerns (Rubin et al. 1999). Whilst well intentioned, we would suggest that these behaviours perpetuate the young person’s perception of social threat. The behaviours will be a source of evidence to the young person that they are less socially able than their peers and thereby maintain their negative attitudes (Ollendick and Hirshfeld-Becker 2002). Parental behaviours will become proxy safety behaviours for the young person. For example, a parent with these beliefs may step in and speak for their child, or give them permission to avoid social situations. These actions will preclude the young person’s opportunities to learn that their fears were unfounded or exaggerated. Some parents become constant companions to their teenage children. This could discourage the child’s peers from approaching or engaging with them, and so directly maintain social isolation.

Whilst some parents may hold overprotective beliefs, other parents may take quite a different view of the social environment and their child. Some parents’ beliefs may emphasise the importance of performing and being heard in a social situation. These may be driven by a parent’s temperamental extraversion or by a socially anxious parent who has coped by relying on impression management safety behaviours. Parents with these views may perceive their child to be missing out on opportunities and as such may express disappointment when their child feels unable to take part in a social commitment (Knappe et al. 2010; Bruch 1989). Likewise, they may push their child to engage in excessively demanding social activities in an attempt to help them overcome their fears. Again, we would suggest that whilst these behaviours are undoubtedly driven by good intentions they will maintain the young person’s anxiety. The young person will interpret expressed disappointment or frustration as evidence of their social failings. When faced with an excessively challenging social scenario, rather than learning new lessons the young person will most likely worry intensely beforehand, rely heavily on safety behaviours to get through the experience, and then ruminate over it afterwards, thereby preserving the negative thinking patterns.

These proposals provide a number of testable hypotheses that have not yet been examined. If supported, the account opens up the potential utility of including specific, focused work with parents in certain cases, where unhelpful parental beliefs and behaviours have been identified and where the young person is not progressing in treatment as one would expect and hope. Several well-established cognitive therapy techniques would be well suited to address parental beliefs and behaviours. For example, parents could be helped to identify unhelpful beliefs they hold and the impact of these on their child’s social anxiety. This could set the stage for behavioural experiments to test out specific predictions. The involvement of the adolescent in these experiments is likely to increase their effectiveness.

### Friendships and Peer Victimisation

As children progress into adolescence, their social relationships become increasingly important (Furman and Buhrmester 1992). They will begin to see their peers as their primary source of social support (Nickerson and Nagle 2005), and so peer relationships take on even greater significance. Adolescents start to manage their own social arrangements, and their relationships will become more complex through this period, with different groups, cliques and subgroups forming, and with the start of romantic relationships. It is unsurprising that good peer relationships are associated with broad indices of well-being in adolescents (Chu et al. 2010). Alden and Taylor (2004) emphasised the importance of interpersonal processes in social anxiety and suggested that social anxiety is maintained by self-perpetuating relationship difficulties.

Numerous studies have examined the relationship between various dimensions of peer relationships and social anxiety cross sectionally. Consistently, less positive peer experiences have been found amongst socially anxious adolescents compared to their less anxious peers, in terms of fewer friends, less peer acceptance, more victimisation and less numerous and happy romantic relationship (see Rubin et al. (2009) for a review). A number of longitudinal studies have considered the impact of social anxiety on later peer relations (e.g. Siegel et al. (2009); Vernberg et al. (1992)). For example, in a two month prospective study examining peer victimisation and social anxiety, Siegel et al. (2009) found that social anxiety predicted relational victimisation, a particular type of peer victimisation. Relational victimisation describes behaviours that use the relationship to in some way harm the intended victim, for example, not inviting a peer to a party, or not allowing another peer to join a group. Turning now to the reciprocal relationship, studies have also examined whether peer processes predict later social anxiety. In line with expectations, more negative peer relations have consistently emerged as a predictor of later social anxiety. For example, in a large sample of 12–19-year olds, lower levels of peer acceptance (as rated by peers) were associated with social anxiety levels one year later (Tillfors et al. 2012). A number of studies have found a relationship between peer victimisation and later social anxiety (Siegel et al. 2009; Storch et al. 2005; Vernberg et al. 1992). Interestingly, two studies (Siegel et al. 2009; Storch et al. 2005) again point to the significance of relational victimisation in social anxiety.

The convergent findings of a reciprocal relationship between social anxiety and peer difficulties are in line with the proposals of Alden and Taylor (2004) that social anxiety may lock individuals into a vicious cycle of interpersonal difficulties. It seems likely that these problems may be heightened during adolescence, when the socially anxious adolescent’s peers are themselves likely to be self-consciousness and particularly sensitive to potential rejection. This leads us to consider how peer problems may be related to the processes specified in the cognitive model. There are a number of ways this might operate, and the following mechanisms are suggested. When an individual experiences peer difficulties this may inform beliefs about their social acceptability directly and drive social anxiety. This suggestion is supported by findings from Grills and Ollendick (2002) in their study of 279 early adolescents. Amongst girls, perception of global self-worth was found to mediate the relationship between anxiety and peer victimisation. Social anxiety will inevitably cause some peer difficulties because gross avoidance of social interactions will limit individuals’ opportunities to forge friendships. It is also suggested that the socially anxious adolescent may be more vulnerable to unfriendly or victimising treatment due to their appraisals of others and the negative beliefs they hold. For example, the tendency for socially anxious adolescents to make negative interpretations of ambiguous social cues may lead them to respond to a fairly neutral situation in an excessively meek, unfriendly or avoidant manner. This may in turn lead to genuinely negative responses from peers. The use of avoidant safety behaviours will contaminate a social interaction; for example, avoiding eye contact and speaking less will convey disinterest. This will make an individual less attractive to their peers, reducing peer acceptance and thereby strengthening negative social beliefs. Furthermore, specific safety behaviours such as agreeing with other people and copying the dress sense of others in order to ‘blend in’ may lead to particular negative responses from peers in adolescence. This is because although adolescence is a time of low resistance to peer influence (Steinberg and Monahan 2007), it is also when individuals are concerned with determining their own identity and they are typically acutely sensitive to being copied.

Delineating these mechanisms provides a wealth of treatment opportunities. For example, the problematic negative thinking patterns and safety behaviours that may be implicated could be targeted well with existing cognitive therapy techniques. However, particular caution may be needed when planning behavioural experiments with teenagers in order to ensure positive outcomes. The ‘pack mentality’ of the social environment, increased frequency of peer victimisation, and heightened sensitivity to peer reactions in this period can all complicate behavioural experiments. Experiments should be set up in social contexts that are likely to lead to positive learning experiences for the adolescent patient. It may be necessary to undertake experiments in analogue social settings prior to testing out fears with known peers.

### Social Media Use

Almost all young people now have access to a smartphone or to a tablet, laptop or desktop computer (Lenhart 2015). Over 90% go online daily, and almost a quarter use the Internet ‘almost constantly’ (Lenhart 2015). Whilst many adolescents spend time playing video games when online, almost all engage with social media. The number of social networking sites has grown in the last few years with the launch of influential sites such as Snapchat and Instagram. Social media is now one of the main ways that young people communicate with one another (Gross 2004), with modes of communication such as email, telephone and SMS falling in popularity. This is in contrast to adults. Although as a group the number of adults using social media has grown over the last few years, age remains negatively correlated with use (Duggan et al. 2015). For this reason, the relationship between social media use and social anxiety is of particularly relevance to adolescents. Because there are relatively few studies that have looked at social media use and social anxiety in adolescents, we have considered studies with both adolescents and young adult samples. Therefore, studies comprising adolescent samples are labelled as such and for those with adult samples we have included details of the age of the sample.

Online communication will hold great appeal for individuals with social anxiety (Pierce 2009). It provides the much-desired opportunity to interact with other people in a less anxiety-provoking setting than a face-to-face interaction (Bonetti et al. 2010; *adolescent sample*). In line with this, a number of studies indicate a positive relationship between social anxiety and time spent on social networking sites (e.g. Lee-Won et al. (2015), mean age = 19.69 years, SD = 1.12 years; Shaw et al. (2015), mean age = 19.2 years, SD = 1.27 years; Orr et al. (2009), mean age = 21.5 years, SD = 5.29 years). One of the reasons online communication may be perceived as less threatening is because individuals feel they are more able to control the information they share and how they present themselves (Caplan 2007; mean age = 19.4 years, SD = 1.37 years). Indeed in a correlational study, adolescents with greater social anxiety reported valuing the controllability of online communication (Peter and Valkenburg 2006; *adolescent sample*). It might be that this is because online, socially anxious individuals are able to engage in safety behaviours more intensively (Harman et al. (2005), *adolescent sample*; Campbell et al. (2006), mean age = 28.7, SD = 10.16 years). For example, they can repeatedly edit posts and spend time preparing responses to messages.

Social anxiety will influence the ways in which people use social media. For example, social anxiety is associated with more passive use of social networking sites such as Facebook (Shaw et al. 2015). Rather than interacting with other users or posting material, people with social anxiety spend more time browsing other peoples’ profiles (Seabrook et al. 2016, *lifespan review, authors note that ‘the majority of studies examined young adults (late teens or early 20s)’*). This type of use will lead individuals to generate unfavourable social comparisons (Vogel et al. 2015) thereby confirming negative social beliefs. Whilst we know that social comparison also occurs offline (Buunk and Gibbons 2007; *review paper*), the online environment may be particular problematic because such a wealth of information is available (for example on profile pages, and through images and videos) and there are fewer limits on how long someone can spend browsing (Vogel et al. 2015; mean age = 18.93 years, SD = 3.94 years). We would also expect social anxiety to influence the ways that socially anxious individuals actively use these sites. For example, it is suggested that when socially anxious individuals do post material, this may tend to be self-denigratory in content; it may be that they would prefer to ‘get the criticism in first’ before they can be maligned by others.

Social media also provides another forum for peer victimisation. ‘Traditional’ bullying may continue after school and extend into online behaviours (Juvonen and Gross 2008; *adolescent sample*). Due to the anonymity that the Internet affords, there may be fewer barriers to perpetrating bullying online and it may be harder to stop (Slonje et al. 2013; *adolescent sample*). A survey of secondary school children found that the most common form of cyber bulling is name-calling via instant messaging (Smith et al. 2008; *adolescent sample*). Cyber bullying is associated with negative outcomes including social anxiety. Cross-sectional studies have found a significant association between social anxiety and cyber bullying (Dempsey et al. 2009, *adolescent sample*; Kowalski and Limber 2013, *adolescent sample*). Two longitudinal studies indicate that social anxiety may confer vulnerability to online victimisation (Juvonen and Gross 2008; van den Eijnden et al. 2014, *adolescent sample*).

When thinking about treatment, we suggest that online social interactions need to be understood and addressed alongside those that occur face-to-face. This would start with a careful assessment of online behaviour at the beginning of treatment. Online safety behaviours could be targeted well with existing cognitive therapy techniques.

## Treatment Implications and Emerging Evidence

Cognitive therapy for social anxiety disorder in adults is comprised of a series of techniques designed to reverse the processes specified in the Clark and Wells cognitive model (1995). The techniques are listed in Table 1. Broadly, the treatment adopts an approach which encourages the patient to discover for him or herself how their social anxiety is maintained. This is achieved through a focus on experiential exercises; for example, a behavioural experiment is undertaken early on in therapy to demonstrate the unhelpful effects of self-focused attention and safety behaviours. Similarly, patients learn to focus their attention externally through a series of practical exercises. We would suggest that this method, of fostering cognitive change through action, is particularly well suited to adolescents, and we expect that many of the core elements of treatment could be employed successfully with adolescents with modest adaptations.

Only two randomised controlled trials have been undertaken examining the effectiveness of therapeutic interventions based on the cognitive model of Clark and Wells (1995) with children and young people. One of these involved children (Melfsen et al. 2011), but we describe it briefly here for interest. Forty-four socially anxious young people aged between 8 and 14 years of age were randomly allocated to individual therapy based on the cognitive model or to a waitlist control group. The authors reported medium to large effects of individual therapy compared to waitlist control on clinician reported (German version of the Anxiety Disorders Interview Schedule; *d* = 0.96) and self-reported outcomes (German version of the SPAI; *d* = 0.91). The results are certainly encouraging. However, the treatment did not represent a full implementation of cognitive therapy. For example, five to six sessions were dedicated to psycho-education. In cognitive therapy we would usually spend no longer than 15 min on this in session one. Critical components, such as the safety behaviour and self-focused attention behavioural experiment that is undertaken in session two of cognitive therapy, were not included. In addition, the trial was with children not adolescents.

Ingul et al. (2014) undertook a randomised controlled trial with socially anxious adolescents in which individual therapy based on the cognitive model was compared to the adolescent group version of Coping Cat (The CAT Project) and an attention placebo. The attention placebo involved group meetings in which socially anxious young people interacted with peers and adults to a similar degree to the treatment arms, but did not receive any of the hypothesised active components of the two treatments. A large effect of individual therapy was found on the SPAI post-treatment (*d* = 2.96). Surprisingly, there was no effect of Group CAT on self-reported SPAI (*d* = − 0.10) and a small effect of attention placebo (*d* = 0.50). The benefits of individual therapy were maintained at follow-up. The results are promising with regards individual therapy; however, treatment was not wholly consistent with cognitive therapy. For example, the first three sessions comprised psycho-education about anxiety, drawing up a broad model of anxiety maintenance, developing an anxiety thermometer and hierarchy, and learning about negative thoughts and thinking errors. An individualised version of the cognitive model was not introduced until session four (compared to session one in cognitive therapy).

In response to these promising findings, we undertook a treatment development case series to test preliminary feasibility of cognitive therapy with adolescents (Leigh and Clark 2016). Cognitive therapy was delivered to five adolescents, all of whom had severe and chronic social anxiety disorder as well as comorbid difficulties at the start of treatment. Four of the five had already received a standard course of CBT without apparent response. By the end of treatment, symptoms of social anxiety, as well as associated anxiety and depression, had reduced to subclinical levels and these gains were maintained at three to six month follow-up. All the young people also showed improved functioning, as evidenced by increased social participation and 100% school attendance at follow-up. Excitingly, we had the first indication that social anxiety treatment may also have a positive impact on classroom concentration, as evidenced by the self-reported improvement across all five patients. The average change (79%) on the primary outcome measure (the Liebowitz Social Anxiety Scale; Liebowitz (1987)) was greater than observed in our trials of cognitive therapy for social anxiety disorder in adults (57 and 63% in Clark et al. 2003, 2006).

Whilst the evidence base is extremely small, the results converge to suggest cognitive therapy may have important promise for adolescents with social anxiety disorder. Notably, although the trial of Ingul et al. (2014) did not test the full treatment, it demonstrated specific treatment effects.

## Conclusions and Future Directions

The present review was motivated by an awareness of the divide in our understanding of the maintenance of social anxiety in adults compared to in adolescents. For example, in adults the development of empirically supported theoretical models to explain the persistence of social anxiety has paved the way for the generation of highly effective NICE recommended cognitive behavioural therapies (see Clark (2013) for a review). In contrast, a detailed mechanistic approach to understanding the maintenance (as opposed to the aetiology) of adolescent social anxiety has largely been lacking. This lack of maintenance models has limited the development of specific treatment techniques for adolescents. In response to this, the present review has focused on one particular model of social anxiety, the cognitive model of Clark and Wells (1995) and its application to social anxiety in adolescents. The review highlights the need for a programme of experimental studies with adolescents in order to test the causal role of the processes specified in the model. Notwithstanding this gap in the literature, the studies reviewed provide very promising support for the application of the model to this age group.

When adopting an adult model such as this for a youth population, it is of course essential to take a developmental perspective. It is suggested that there will be developmental influences on the psychological processes specified in the adult model. For example, as individuals progress through adolescence we would expect that they may use different safety behaviours (younger adolescents may rely on parents to speak for them but this may become developmentally inappropriate for older adolescents) and that the safety behaviours they tend to rely on may change over time (such as an increasing use of more sophisticated impression management safety behaviours with age). Turning to mental imagery, given that the ability to generate, inspect, maintain and manipulate mental images develops in an extended fashion through childhood and adolescence (Burnett Heyes et al. 2013), socially anxious adolescents may be particularly vulnerable to distressing social imagery, finding it difficult to inhibit images or shift attention away from them when they occur. But given this susceptibility, adolescents may also be particularly responsive to interventions targeting negative imagery. Likewise, given that adolescence is associated with heightened self-consciousness, socially anxious adolescents may show especially intense self-focus in social interactions, but they may also be especially responsive to an attention training intervention. No studies have yet examined these questions. As well as considering the developmental influences of the processes specified in the adult model, the explanatory power of the model will be enhanced with the addition of processes that are particular to adolescents, such as parental and peer processes. When considering these factors, it will be important to specify the mechanisms by which they maintain social anxiety in order to develop specific interventions.

The present review underscores the great potential for adapting and refining the cognitive model of social anxiety disorder (Clark and Wells 1995) for adolescents in order to improve treatment outcomes for this population.

## Footnotes

1 The term ‘correlational’ is used to refer to observational studies that measure co-variation either within or between groups but do not experimentally manipulate the variable.
